# A Bayesian approach to comparing common models of life-course epidemiology

**DOI:** 10.1093/ije/dyab073

**Published:** 2021-05-10

**Authors:** Justin Chumbley, Wenjia Xu, Cecilia Potente, Kathleen M Harris, Michael Shanahan

**Affiliations:** 1 Jacobs Center for Productive Youth Development, University of Zurich, Zurich, Switzerland; 2 University of North Carolina at Chapel Hill, Carolina Population Center, Chapel Hill, NC, USA

**Keywords:** Bayesian, life course, parameter ranking, developmental

## Abstract

**Background:**

Life-course epidemiology studies people’s health over long periods, treating repeated measures of their experiences (usually risk factors) as predictors or causes of subsequent morbidity and mortality. Three hypotheses or models often guide the analyst in assessing these sequential risks: the *accumulation* model (all measurement occasions are equally important for predicting the outcome), the *critical period* model (only one occasion is important) and the *sensitive periods* model (a catch-all model for any other pattern of temporal dependence).

**Methods:**

We propose a Bayesian omnibus test of these three composite models, as well as *post hoc* decompositions that identify their best respective sub-models. We test the approach via simulations, before presenting an empirical example that relates five sequential measurements of body weight to an RNAseq measure of colorectal-cancer disposition.

**Results:**

The approach correctly identifies the life-course model under which the data were simulated. Our empirical cohort study indicated with >90% probability that colorectal-cancer disposition reflected a sensitive process, with current weight being most important but prior body weight also playing a role.

**Conclusions:**

The Bayesian methods we present allow precise inferences about the probability of life-course models given the data and are applicable in realistic scenarios involving causal analysis and missing data.

**Key Messages**

- Life-course epidemiological methods often test or compare models via points in parameter space.
- Instead, we propose using *regions of practical equivalence* for both model comparison and model decomposition.
- In particular, we describe the first general method for coherently decomposing the broad ‘sensitive model’ into scientifically meaningful sub-models.

## Introduction

Life-course epidemiology typically examines associations between repeated measures of exposures and subsequent health over many decades of life. This approach has proven popular in the study of chronic forms of morbidity, which often involve multiple exposures over different developmental stages (hereafter ‘measurement occasions’) and an appreciable latency period between exposure and outcome.^1^ Research in this area is generally guided by three life-course models or hypotheses:^2^ the accumulation model, whereby multiple exposures of roughly equal importance predict an outcome; the critical period model, whereby one of multiple exposures (often very early in development) is decisively important; and the sensitive period model, whereby several exposures predict the outcome in non-trivial ways. Even though these models greatly simplify life-course processes that are likely very complex, they have nonetheless proven influential and useful in the study of health. Adjudicating among these models—as well as several others that are relevant in certain cases^3^—is thus a fundamental task in life-course epidemiology.

Two main approaches have been proposed in the literature: a structured approach^4–6^ and a Bayesian approach.^7^ The structured approach compares several nested models to a saturated model. Goodness-of-fit criteria are then used to ascertain the model that best fits the observed data. The Bayesian approach readily accommodates previous knowledge and arguably improves scientific interpretability,^7–12^ but is still in its infancy.

Madathil *et al.*^7^ proposed certain Bayesian procedures for contrasting the critical, accumulation and sensitive models given an observed data set. Their procedures are based on a non-linear transformation of the linear parameter $\theta =(\theta_{1},..,\theta_{T})$ in a generalized linear model of outcome Y on the subject’s exposure history $x=(x_{1},..,x_{T})$ over T periods. In particular, they estimate θ=δw where scalar δ captures ‘the total lifetime effect’ and w is a set of *proportions* encoding the relative effect of each measurement occasion. Madathil *et al.*^7^ then seek an alternative to conventional model selection by studying distance distributions and credible sets of posterior p(w|y). Some implicit assumptions of this approach are outlined in the Supplementary Material, available as Supplementary data at *IJE* online.

**Figure 1:**
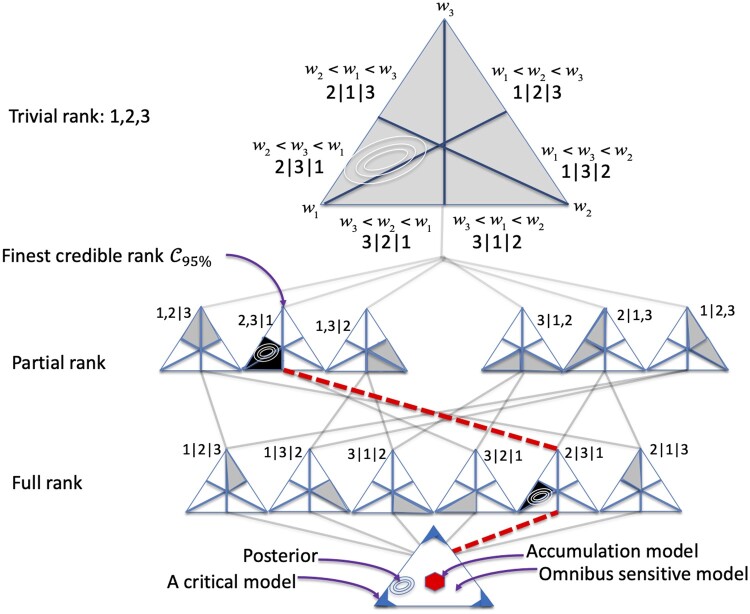
This graphic depicts our proposed method by considering the posterior distribution of a schematic three-life-period model (for ease of presentation) on $\Delta^{3}$. The diagram reads bottom to top. The schematic posterior probability iso-contours are depicted inside the simplex. Starting at the base, we first evaluate posterior mass on the omnibus sensitive, critical and accumulation models using ϕ. Specifically, we chose thresholds *a=*0.15*, b =* 1- a for the univariate range, which together imply the polygonal regions of practical equivalence (ROPEs) in the simplex at the base of the figure. (The red central region corresponds to the interval [0, *a*] = [0, 0.15], the blue corner regions correspond to the interval [*b*, 0] = [0.85, 1] and the non-shaded, intermediate region corresponds to the interval (*a, b*) = (0.15, 0.85). See Supplementary Material, available as Supplementary data at *IJE* online, for a higher-resolution image and critique.) Our schematic posterior mass does not lie in the center or in a corner, but is consistent with the intermediary sensitive model, motivating us to ask ‘*which* specific sensitive sub-model?’ We answer this question by advancing up the diagram (choosing the most probable event at the next level up). Note that upward paths correspond to the subset inclusion relation ⊆, so posterior probability monotonically increases accordingly: see Figure 2 for a numerical example. We stop at the first region with probability >90% and call this region the 90% finest credible region. In our schematic example, this is 2,3|1. This ranking procedure would have been inconclusive if (and only if) the first such region is the vacuous ranking at the top of the diagram. Now suppose that, from the base of the diagram, our posterior had instead supported the critical model. Then, our follow-up decomposition could identify *which* critical period by simply examining $p(w_{i}>t|y)$ for some high threshold t. Finally, had we chosen the accumulation model at the base of the diagram, no further decomposition would have been required.

In this paper, we introduce alternatives for summarizing the posterior distribution p(w|y) of Madathil *et al.*^7^ and many related life-course models. In particular, we compare the critical, accumulation and sensitive models and sub-models by their posterior probability, rather than using complicated credible sets or the density of distances from competing point hypotheses^7^ (see the base of Figure 1). Our approach is designed for situations of relatively high scientific uncertainty about which model is true. For example, without knowing which specific period may be critical in advance, we must examine all the critical models without succumbing to a multiple-comparisons problem.

Unlike classical omnibus tests, e.g. F-tests or ANOVA, however, we simply calculate the probability of three predefined regions of parameter space. Each region uniquely characterizes one model: it is the model’s ‘region of practical equivalence’ (ROPE). In detail, our proposed composite test involves first calculating the greatest difference between the weights of any two periods, i.e. the range of component parameters $w=(w_{1},..,w_{T})$. This range characterizes the three models: it equals 0 and 1 for the accumulation and critical models, respectively, and sits between these extremes for any sensitive model. In other words, the (univariate) range contains all the information required to construct polygonal regions of practical equivalence (ROPEs) for the three broad life-course models (see the base of Figure 1).

After assessing the composite models, *post hoc* decompositions provide more detail. The critical model can very easily be decomposed by just examining the posterior components of $w_{i}$ because exactly one will have high mass on high values, say $w_{i}>0.85$. Crucially, we show that the sensitive model also decomposes nicely via a simple univariate construction, this time via *ranking* measurement occasions 1,..,T by importance, i.e. which are more ‘sensitive’ (have larger weights) than other time points. A simple algorithm then yields a concise conclusion such as ‘with 90% posterior credibility the first two measurement occasions matter more than any subsequent occasions’. For reasons discussed below, we call this latter quantity the ‘finest credible rank’. The final accumulation model requires no decomposition: there is only one way for all weights to be (approximately) equal.

Whereas our goal here is not to improve causal identification per se, our proposed methods are general enough to be broadly applied in causal and non-causal frameworks. With or without causal identifiability, it is often important to rank parameters or to conclude that they are unrankable or tied (e.g. purely descriptive research). For simplicity, we therefore demonstrate our methods via the simple model that Madathil *et al.* (2018) introduced above (where strong assumption of no feedback between time-varying treatments and time-varying confounders would be required to achieve causal identifiability). In the discussion, we explain how our methods apply under weaker identifiability conditions, non-linear models and models without a simplicial parameter space.

The paper is structured as follows. We first describe the mathematical rationale for our approach and then explain the goals and specifications of our simulation study. Having established the statistical validity of our approach, we apply our methods to an empirical example relating pronounced body weight over the life course to colorectal-cancer disposition. The discussion then outlines the strengths and limitations of our approach.

## Methods

### Rationale

Figure 1 provides an overview of our proposed methodological strategy. Assuming the model of Madathil *et al.*,^7^ our generalized linear parameter is taken to be θ=δw, where w is a vector whose components are proportions satisfying $w_{t}\geq 0,\sum_{t}w_{t}=1$. Thus, w belongs to the *T*-part simplex, which we denote as $\Delta^{T}$. Components of $w=(w_{1},..,w_{T})$ usefully capture the *relative* importance of exposure x at each measurement occasion t=1,..,T for predicting outcome Y. Conversely, δ captures ‘the total lifetime effect’.

We define our composite test by choosing two thresholds a,b∈[0,1] to partition the values of ϕ into three intervals, which are practically equivalent to the accumulation=[0,a], sensitive=(a,b) and critical=[b,1] models. These intervals encode competing (non-overlapping) models that are exhaustive: p(sensitive or accumulation or critical|y)=1. We approximate the posterior probability of these three life-course models by the fraction of posterior Markov Chain Monte Carlo (MCMC) samples falling within the *accumulation*, *critical* and *sensitive* intervals, respectively. We then conclude which model is most credible. For example, the sensitive model may be deemed credible in absolute terms if >90% of the posterior samples of ϕ|y fall within the *sensitive* interval. Or it may be deemed most credible in relative terms if the estimated Bayes factor

$$BF_{\mathrm{sensitive}}=\;\frac{p(\mathrm{sensitive}|y)/p(\mathrm{sensitive})}{p(\mathrm{critical}\;or\;\mathrm{acculumulation}|y)/p(\mathrm{critical}\;or\;\mathrm{accumulation})}$$

exceeds some threshold.

The ROPEs (*a, b*) and [*b*, 1] above encode ‘composite models’. The posterior mass on [*b*, 1] assesses the global critical model, i.e. is *any* of T critical periods plausible? Whenever this model is plausible, it is trivial to assess *which* component is critical: simply inspect the individual components of *w* because exactly one will markedly deviate from zero. Similarly, the sensitive model (*a, b*) encompasses *all possible full rankings*. Decomposing this latter is more tricky and leads us to now define the concept of ‘finest credible rank’.

Let the function f label each point w with its full ranking. For example, f gives the point w=(0.2,0.7,0.1) the label 3|1|2 because the third measurement occasion is the least important, followed by the first occasion, with the second occasion being the most important. This notation for labelling full rankings is adapted from that of Lebanon and Mao.^13^ The full set of T! full rankings comprise mutually exclusive sub-models of the sensitive model. We again approximate the posterior probability of these full rankings by the fraction of MCMC samples satisfying the relevant inequalities ($w_{3}<w_{1}<w_{2}$ in this example). Thus, we can assess whether the most probable full ranking of measurement occasions by importance is e.g. 3|1|2 or whether p('3|1|2'|y)≥0.90. This assessment provides insights into our multivariate posterior p(w|y) without the inconvenience of constructing complicated continuous multivariate credible sets.

Importantly, the notation extends to coarser, *partial rankings*, such as 3,1|2, and calculates their posterior probability. A partial ranking can be viewed as a collection of full rankings: this ambiguity means that they carry less information about the relative importance of measurement occasions. For example, the partial ranking 3,1|2 (equivalently denoted as 1,3|2) represents all points w that can be ranked as *either* $w_{3}<w_{1}<w_{2}$ or $w_{1}<w_{3}<w_{2}$: it, therefore, encodes points w for which $w_{2}$ is unambiguously the most important period. Then, if p('1,3|2'|y)≥0.90, we can say with 90% credibility that the second measurement occasion is most important, even though we can say nothing about the relative importance of the first and third occasions. The proposed algorithm starts at the maximum full ranking *a posteriori*, then recursively seeks the maximum *a posteriori* ranking at the next level of resolution (among all partial rankings with one additional bar ‘|’ exchanged for a ‘,’). This is depicted in Figure 1 and gives the optimal nested sequence of sub-models of the sensitive model. We define the β% ‘finest credible rank’ (FCR), denoted as $C_{\beta }$, as the first (finest, or most informative) such partial ranking with β% posterior probability. This is the most informative statement that can confidently be made about the relative sensitivities of different measurement occasions.

**Figure 2:**
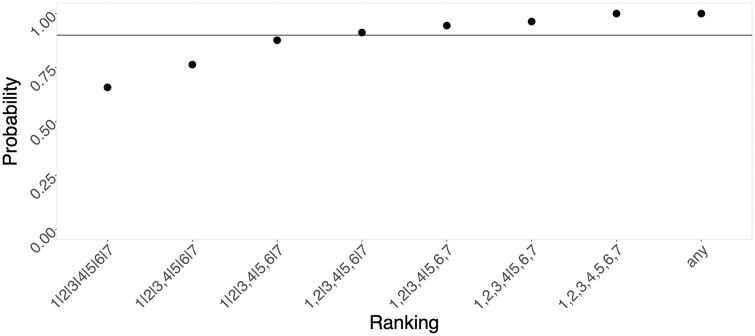
Posterior cumulative density over increasingly coarse partial rankings. The ground truth in this example was 1|2|3|4|5|6|7. Progressing from left to right across the *x*-axis, rankings become coarser by the loss of one distinction (‘|’). All points above the horizontal black line have ≥90% posterior credibility.

Figure 2 illustrates this recursive scheme for whittling down the sensitive model using a hypothetical numerical example. It illustrates inference from data where we know the true full ranking was 1|2|3|4|5|6|7. The figure gives the ‘cumulative density function’ of the best nested sequence of subsets of $\Delta^{T}$. Among these, $C_{90\%}$ is the finest 90% credible ranking (represented by the fourth point from the left in Figure 1). The candidate partial ranking at each step in the sequence from left to right is the most credible (maximum probability) coarsening of the preceding candidate. In this example, the partial ranking 1,2|3,4|5,6|7 is the finest ranking with 90% credibility. That is, 1,2|3,4|5,6|7 is the 90% FCR: we conclude that seventh measurement occasion has the greatest weight, the fifth and sixth occasions (whose relative importance cannot be differentiated) make the next largest contribution to explaining the outcome variation, followed by the third and fourth occasions (again indistinguishable in terms of importance) and finally the first and second occasions. Thus, in this hypothetical example, we can be confident of a coarse pattern of increasing sensitivity of outcome to exposures later in life. However, we are not entirely confident that sensitivity is fully monotonically ordered: this conclusion warrants only ∼70% credibility as given by the probability of the left-most entry on the *x*-axis, the full ranking 1|2|3|4|5|6|7.

As our discussion of Figure 2 illustrates, we require high posterior precision to faithfully identify the true full underlying ranking, such that our 90% FCR is a *full* ranking. When posterior uncertainty is higher, we can only partially infer the parameter ranking, i.e. the 90% finest credible interval will be a *partial ranking* (possibly even the trivial ranking). This can also be pictured by contracting or expanding the isocontours in our schematic posterior density in Figure 1.

A detailed discussion of alternative priors on ϕ and f is presented in the Supplementary Material, available as Supplementary data at *IJE* online.

### Goals of the simulation

We ask whether the univariate credible interval for δ|y appropriately excluded zero, i.e. whether it correctly inferred whether *any* time period is relevant for the outcome. If the answer is positive, it makes sense to broadly examine the three competing models (critical, accumulation and sensitive) via the posterior distribution ϕ|y. If ϕ|y additionally supports a sensitive model, f|y and the probable ordering of weights should be examined. Therefore, the objectives of the simulation study were (i) to assess whether δ|y appropriately excluded zero and, if so, (ii) to assess whether the range ϕ|y successfully discriminates between the accumulation, critical and sensitive models, and, in the case of the last possibility, (iii) to assess whether rank f|y succeeds in identifying the correct ranking of time periods by their sensitivity, as discussed next.

Knowing the simulated ground truth $w^{*}$ and its corresponding true full ranking $f(w^{*})$, our principle questions concern its relation to the inferred *finest* β*% credible ranking*, which we denote as $C_{\beta }$:

- Is the FCR $C_{\beta }$ consistent with the true full ranking? We say it is inconsistent if there is any i≠j for which the FCR asserts $w_{i}<w_{j}$ whereas, in fact, $w_{j}<w_{i}$ in the simulated ground truth. Otherwise, we say it is consistent.
- How much ‘information’ does $C_{\beta }$ retain? Here, we use $q=r/r^{*}$ with values between 0 and 1 to measure the quality of information in $C_{\beta }$, where r is the number of distinctions (inequalities or bars ‘|’) in $C_{\beta }$ and $r^{*}$ is the true number in $f(w^{*})$. Therefore, a larger q means a more informative inference.

The first question expresses the minimal requirement that $C_{\beta }$ does not contradict the truth. The second question is motivated by the desire for $C_{\beta }$ to be as informative as possible. Ideally, it should faithfully retain *all* distinctions made in the true ranking $f(w^{*})$.

### Simulation parameters

Our simulation fully reproduced and extended that of Madathil *et al.*.^7^ Namely, we simulated a three-period life-course study, assuming no measurement error in the variables. In particular, for participant i, we sampled three Gaussian exposure variables $x_{i}=(x_{1},x_{2},x_{3})$ with a correlation of 0.7 and 0.49 between adjacent and non- adjacent measures, respectively. Data sets were simulated for all combinations of the four life-course models (the two distinct sensitive models defined below, in addition to the accumulation and critical model) and three sample sizes (n=700, 1500, 3000). The ground-truth weight values of the simulation, denoted with an asterisk ‘*’, were as follows: (i) pure accumulation model $w_{i}^{*}=(\frac{1}{3},\frac{1}{3},\frac{1}{3})$; (ii) linear sensitive period model with weights $w_{ii}^{*}=\frac{1}{1+2+3}(1,2,3)$; (iii) first and second measurement occasions as a sensitive period $w_{\mathrm{iii}}^{*}=(0.75,0.2,0.05)$; (iv) third measurement occasion as a critical period $w_{iv}^{*}=(0,0,1)$.

We then extended the above three-period simulation to five and seven periods as follows: the accumulation model (i) above was generally $w_{i}^{*}=(\frac{1}{T},..,\frac{1}{T})$; (ii) was generalized in the obvious way to $w_{ii}^{*}=\frac{1}{\sum_{i=1}^{T}t}(1,2,..,T)$; and (iii) and (iv) were padded with zeros, e.g. $w_{\mathrm{iii}}^{*}=(0.75,0.2,0.05,0,0,0,0)$ and $w_{iv}^{*}=(0,0,0,0,0,0,1)$, respectively, in seven dimensions.

We independently varied the lifetime effect $\delta^{*}$ between 0, 1 and 2. The values simulate situations where a unit change in the total exposure (or weighted average exposure) over all time points increases the outcome by 0, 1 or 2 units. These fixed underlying settings (estimands) are again denoted as ‘*’, to distinguish them from their posterior inferred counterparts. Given $\delta^{*}$ and $w^{*}$, we then generated $y_{i}=\delta^{*}\sum_{j=1}^{T}x_{ij}w_{j}^{*}+\epsilon_{i}$ with independent $\epsilon_{i}∼N(0,1)$, for i=1,..,n.

### The prior distribution and inference

In accordance with the data-generating model above, we used Bayesian linear regression for inference. For the moment, we follow Madathil *et al.*^7^ in their choice of a uniform prior over all $\Delta^{T}$, i.e. a non-informative Dirichlet prior for weights p(w)=Dirichlet(w|1), where 1 is a vector of T ones. In cases in which there is plausible justification for bias towards the accumulation or critical models, the hyperparameter can be generalized to α1, with α>0. Then, it is well known from the properties of the Dirichlet that choosing α<1 implies a prior bias toward the critical model and α>1 implies a prior bias towards accumulation. We revisit these options below. We also chose a weakly informative Cauchy prior for the lifetime effect p(δ)=Cauchy(δ|0,2.5).

To summarize, the model was

$$\begin{array}{c} y_{i}=\delta \sum_{j=1}^{T}x_{ij}w_{j}+\epsilon_{i}\\ \epsilon_{i}\overset{i.i.d}{∼}n\mathrm{ormal}\left(0,\sigma \right)\\ \delta ∼\mathrm{Cauchy}\left(0,\;2.5\right)\\ w∼\mathrm{Dir}\left(\frac{1}{D},\ldots ,\frac{1}{D}\right)∼\;\mathrm{uniform}\left(\Delta^{T}\right)\\ \;\sigma ∼\mathrm{lognormal}(1,1) \end{array}$$

The Hamiltonian MCMC sampler implemented in Stan^14^ was used to acquire 50k marginal posterior samples of δ|y and w|y. After performing standard convergence tests, we examined δ|y and derived ϕ|y and f|y by applying ϕ,f to each point in our posterior sample w|y.

## Results of simulation

### Posterior lifetime effect δ and range ϕ

In every simulation, the univariate credible interval for δ|y appropriately included/excluded zero. This pattern suggests that δ|y is a faithful omnibus measure of lifetime effect. In simulations with evidence of non-zero δ|y, we used ϕ|y to adjudicate between the critical, accumulation and sensitive models.

By choosing two thresholds a=0.15 and b=1-a in the support of ϕ|y, we constructed three intervals ‘practically equivalent’ to the accumulation, critical period and sensitive models, as discussed in the ‘Rationale’ section. Table 1 reports the resulting confusion matrix, which relates the inferred models (column variable) to the underlying truth (row variable). We made a conclusive choice between these cases whenever one of them had >0.9 posterior probability. Otherwise, our inference was considered inconclusive/unknown (u). There were 9/72 inconclusive inferences. Inconclusive inferences reflect posterior uncertainty, which, in turn, reflects either small effects or sample sizes.

**Table 1:** Confusion matrix for true and inferred life-course models

	a	s	c	u
i. a	14	0	0	4
ii. s	0	18	0	0
iii. s	0	17	0	1
iv. c	0	0	14	4

Rows refer to the true models: i. accumulation; ii. linear sensitivity; iii. non-linear sensitivity; and iv. critical period. Columns refer to the inferred models: accumulation (a), sensitive (s) and critical (c) hypotheses, as well as simulations with evidence of a non-zero lifetime effect and inconclusive/unknown (u).

However, all the conclusive results in our simulation study correctly identify the ground truth. In general, there were no incorrect inferences in our study, such as would arise if, for instance, we concluded that the accumulation model was true when in fact data were generated under the sensitive model. For example, of the 18 simulations in which the accumulation model was true (Table 1, row 1), inference was correct in 14 cases and inconclusive in 4 cases. This table shows that ∼90% (63/72) of inferences were both conclusive and correct.

The results of Table 1 were qualitatively similar when we instead set (a,b)=(0.1,0.9) or (a,b)=(0.2, 0.8). In complementary analyses, we also used a conventional 3.2 threshold for the (log) Bayes factors discussed in ‘Rationale’ above (estimated by the ratio of prior to posterior samples in each ROPE). Results were again qualitatively similar, albeit with slightly inferior performance: there were still no misclassifications, but more inconclusive results (more false-negative Bayes factors below the threshold of 3.2).

### Posterior FCR $C_{\beta }$

When the sensitive model was credible (i.e. the 18 + 17 cases in column s of Table 1), it made sense to ask which periods were more or less sensitive. For these cases, we calculated the finest partial ranking $C_{\beta }$ of parameters.

Regarding the goals of the simulation, we found that

- the inferred finest partial ranking $C_{\beta }$ never violated the ground truth; and
- on average, over all simulations, ∼0.71% of the distinctions were preserved. Table 2 shows that q, the proportion of distinctions preserved in $C_{\beta }$, increased with the simulated sample size. On average, over simulations with the lowest sample of 700, 52% of the distinctions were preserved. This increased to 72% and 89% with higher sample sizes. This pattern shows how increased posterior precision in w|y translates into increased precision of the inferred rank.

**Table 2:** Simulated sample sizes and mean proportions *q* of distinctions preserved by the posterior credible ranking

*N*	*q*
700	0.52
1500	0.72
3000	0.89

The quantity *q* refers to the proportion of distinctions in the ordering of the true underlying parameter which are preserved in the inferred ordering or FCR.

## Empirical example

### Data and regression model

The data are from the National Longitudinal Study of Adolescent to Adult Health (Add Health), a representative study of US adolescents in Grades 7–12 during 1994–1995 who were followed into adulthood over five waves of data collection.^15^ Specifically, the present study combines body-weight data from (i) parental report of birthweight, (ii) Waves I and II (12–20 years), (iii) Wave III (18–27 years), (iv) Wave IV (25–33 years) and (v) Wave V, Sample 1 (33–42 years).

During Wave V, RNAseq abundance data from peripheral blood samples were collected (currently, *n* = 1132 samples collected in the 2016–2017 window have been fully preprocessed; details of data collection and preprocessing can be found in the Supplementary Material, available as Supplementary data at *IJE* online). Our outcome variable was a scalar mRNA colorectal-cancer signature constructed as the normalized, weighted mean of 127 upregulated genes (given positive weights) and 2 downregulated genes (given negative weights). These genes are collectively implicated in colorectal-cancer biology.^16^ Normalization was performed using a reference-gene normalization procedure^17^ that converts raw counts onto a log scale. We had both mRNA and phenotype data for 893 participants. For participant i, we denote the ‘pronounced body weight’ dummies on the five measurement occasions $x_{i}=(x_{1},x_{2},x_{3},x_{4},x_{5})'$ and their real-valued mRNA score ${\bar{y}}_{i}$. Our models controlled for biological sex, race/ethnicity, age at the time of the survey during Wave I, preterm birth status, region and sample-specific quality-control measures for mRNA. For notational simplicity, these control variables are denoted by the vector c in the equations below.

The goal was to predict a pre-symptomatic mRNA colorectal-cancer signature from five measurements of pronounced body weight, taken on five separate measurement occasions: pronounced birthweight (defined as a birthweight >8.8 pounds or <5.5 pounds) and then pronounced body weight [body mass index (BMI) ≥ 30] at Waves I–V. We wanted to know whether one measurement occasion was *critical* for predicting variation in this cancer signature, whether all occasions mattered equally (*accumulation*) or whether multiple occasions mattered to different degrees (*sensitive*). In the last case, we sought a credible and informative statement about the relative importance of the measurement occasions.

A Bayesian MCMC approach was used to estimate the reparameterized regression model of Madathil *et al.*^7^ as ${\bar{y}}_{i}=\alpha +\delta \sum_{i=1}^{5}w_{i}x_{i}+c'\gamma +\epsilon_{i}$. Here, α is an intercept, δ and $w=(w_{1},..,w_{5})$ are as defined throughout this work and γ is the regression parameter vector for our control variables. The priors were the same as for the simulations above, with the addition that $\gamma_{i}∼\mathrm{Cauchy}(0,2.5)$ independently for each control variable. Thus, assuming a uniform prior distribution on w and weakly informative normal prior distributions on δ and γ, MCMC was used to collect 50k samples from their posterior distributions using the R package RStan, as implemented by Guo *et al*.^18^ In particular, we pooled post-burn in samples from five independent chains of 10k after performing standard diagnostics to ensure convergence (we ran five independent parallel Hamiltonian Monte Carlo chains, each with 20k iterations, and convergence was confirmed by checking trace plots and Rhat values).

## Results

The posterior mean weight vector was *w* = (0.174, 0.104, 0.055, 0.077, 0.590). The posterior mean δ was 0.14 with 95% credible interval (0.09, 0.19). The central 90% and 95% posterior intervals for the lifetime effect δ were [0.1, 0.18] and [0.09, 0.19], respectively. These exclude zero, so we proceeded to evaluate the suitability of the accumulation, critical and sensitive models. We did this by choosing two thresholds a=0.15,b=0.85 to partition the posterior ϕ|y into three intervals (recalling that ϕ is defined as the difference between the largest and smallest components of w).

The posterior probabilities for the accumulation, sensitive and critical models were 0.0004, 0.9973 and 0.0023, respectively. The sensitive model was thus the most credible, warranting an examination of the relative importance of the measurement occasions by examining the probable ranking of the weights. Table 3 presents the highest-probability partial rankings at each level of granularity. The most probable *full* ranking was 3|4|2|1|5. However, this highly informative statement carried only 19.7% posterior credibility. In fact, the 90% finest credible ranking, with 94.1% credibility, was 3,4,2,1|5, which can equally be written as 1,2,3,4|5 in our notation (because, in our notation, the order of numbers *between* any two adjacent bars is arbitrary). Thus, the final (fifth) measurement occasion matters more than any other (birth or Waves I–IV) in predicting the outcome. We have already concluded that (some of) these earlier periods probably matter: the posterior probability that they all have a weight of zero (i.e. the probability of a *critical* pattern of weights) is only 0.0023. Yet, our inferential uncertainty in this data set is too high to warrant any further claims about the relative importance of these earlier measurement occasions. It should also be noted that, with 74% credibility, Wave I matters more for colorectal disposition than Waves II–IV, but less than Wave V.

**Table 3:** Ranking measurement occasions by their importance for colorectal cancer (i.e. their relative magnitude)

Ranking	Probability
3|4|2|1|5	0.197
3|4,2|1|5	0.350
3,4,2|1|5	0.737
3,4,2,1|5	0.941
3,4,2,1,5	1.000

The best sequence of nested sub-models (partial rankings) of the sensitive model and their posterior probability.

## Discussion

Our approach builds on Madathil *et al.*’s^7^ proposal to use a Bayesian framework to evaluate different models of life-course epidemiology. Like those authors, we pursue a detailed posterior analysis of a single model under weakly informative priors. We propose Bayesian composite tests and decomposition procedures based on the posterior probability of the critical, accumulation and sensitive models and sub-models. Importantly, our approach relaxes dependence on continuous multivariate confidence sets, distance densities and the requirement to specify point hypotheses (‘expected weight vectors’).^7^ Instead, each model is identified with a region or ‘ROPE’ of parameter space $\Delta^{T}$, and we simply evaluate the posterior probability of that region. Our two-fold approach therefore simplifies the comparison of common models of life-course epidemiology.

Our simulations illustrated that our methods were not capricious: adjudicating between the composite life-course models never pointed to the wrong model and model decomposition rarely confused the relative importance of measurement occasions. Therefore, we studied an empirical data example examining associations between an mRNA-based cancer signature and body-weight history using Add Health cohort data. Our empirical example was motivated by the fact that high BMI is associated with a high risk of colorectal cancer,^19^^,^^20^ which is among the most common cancers and a leading cause of cancer death.^21^ However, the role of BMI at different points in the life course has not been examined. Our method concluded that the critical and accumulation models do not offer a compelling explanation for the data. Instead, a sensitive pattern emerged in which contemporary pronounced body-weight status was most salient to the abundance levels of mRNA for genes associated with colorectal cancer and pronounced body weight at earlier ages (and possibly high/low birthweight status) was also independently predictive. Such findings, combined with prior studies,^22^ support the need for further study with larger samples that include data on life-course patterns of body mass.

Our proposed methodological approach may be particularly attractive in situations of high prior uncertainty about the true relative importance of different measurement occasions, which is often the case in life-course epidemiology. Assuming that the sensitive model (or critical model) is true, there may be no compelling reason to hypothesize one particular pattern of temporal sensitivity to exposure over another. In such cases, it often makes sense to place a uniform distribution over parameter space $\Delta^{T}$ and, therefore, over different patterns of sensitivity or criticality. Our sensitive decomposition relies on the (component-wise) *order* of the parameters. A precursor of this idea appears in Table 2 of Madathil *et al.*,^23^ albeit on an *ad hoc* subset of sensitive models (see also Madathil *et al.*^24^). Our work formalizes this idea into an algorithm that explicitly optimizes over the set of all possible decompositions (rankings), while controlling multiple comparisons. We have achieved this by rigorously defining and assessing the error, and information content, of an inferred decomposition (see ‘Goals of the simulation’). The result is a method that complements other common summaries of the posterior distribution (e.g. mean and median).

Our methods have some limitations. First, our approach is unsuitable when exposures are measured at many, closely spaced time points.^25^ However, alternatives are suggested by Madathil *et al.*^7^ Second, the posterior precision of our methods requires relatively large sample sizes (as discussed in the ‘Results’ section) and, therefore, may be best suited to large epidemiological panels. Third, our simple expository model is too restrictive for many applications. For example, it is essentially linear in the parameters and assumes without justification that all coefficients have the same sign. Neither can its parameters benefit from a causal interpretation except under quite strong assumptions.^26^^,^^27^ Fourth, assuming that we are happy with the broad geometry of our ROPEs (see Supplementary Material, available as Supplementary data at *IJE* online, for a critique and discussion), there remains the question of how to set threshold values a, b to define our composite tests. In what follows, we discuss and seek to resolve these last two concerns.

Despite the attractive parameterization that we have adopted from Madathil *et al.*,^7^ their model is not explicitly causal or longitudinal: it has the same limitations and explanatory power as a generalized linear model on the restricted parameter space $R_{\mp }^{T}=\pm (0,\infty {)}^{T}\subseteq R^{T}$. In this restricted space $R_{\mp }^{T}$, all components of any parameter vector must have the same sign (i.e. each measurement occasion affects the outcome in the same direction). For example, in the 2D case $R_{\mp }^{2}$, this region is simply the union of the positive and negative quadrants. But we can easily promote this untested assumption to an explicit model, with posterior probability given by $p(R_{\mp }^{T}|y)$ under a standard, *unrestricted* or ‘encompassing’ Bayesian generalized linear model with parameters anywhere in $R^{T}$. Namely, with a lower computational cost, we can simply estimate an unrestricted Bayesian generalized linear model and assess the proportion of posterior samples in $R_{\mp }^{T}$.

Moreover, we can also evaluate the two halves of this union separately: so $p(R_{+}^{T}|y)$, for example, examines the more specific claim that all components are positive, i.e. δ>0 in the parameterization of Madathil *et al.*^7^ Given posterior evidence for one quadrant (e.g. $R_{+}^{T}$), a simple transformation $\theta_{i}\mapsto \theta_{i}/\sum_{i=1}^{T}\theta_{i}$ from this quadrant takes us back to the simplex $\Delta^{D}$, where all our proposed methods again apply. This observation indicates that our proposed methods apply in any model with a parameter vector, not the model of Madathil *et al.*^7^ In fact, our procedure for identifying the FCR does not itself require the transformation $\theta_{i}\mapsto \theta_{i}/\sum_{i=1}^{T}\theta_{i}$ and can be applied directly to the encompassing vector space $R^{T}$. Similarly, ϕ=0 characterizes the equality or accumulation model in both $R^{T}$ or $\Delta^{T}$, depending on which is most convenient. The accumulation model can therefore always be assessed by measuring the posterior mass of ϕ|y within a ROPE around zero.

In summary, our methods apply generically in the sense of not requiring many modelling assumptions, such as parameter linearity, error independence (e.g. temporal correlations) or strong causal identification conditions. They consequently apply to lists of real-valued parameters in one’s favourite epidemiological model, to variance components of a multilevel model, etc. For example, the parametric g-formula is amenable to a Bayesian approach, e.g. Keil *et al.*^28^ Furthermore, our methods can be combined with any procedure that addresses missing data, so long as this procedure returns a well-defined posterior distribution. For example, one may mix draws from the posterior distributions of each multiply-imputed (complete) data set (e.g. Zhou and Reiter^29^).

We now consider how to set threshold values a,b. This specification is important partly because it affects the prior probability of our three composite models. One might adjust for this prior influence via relative measures such as p(sensitive|y)/p(sensitive) or the Bayes factors discussed in our ‘Rationale’. But these statistical solutions do not free us from considering the scientific meaning of our models. The generic—and unhelpful—guidance is to ensure that the model ROPEs capture the scientific meaning of ‘practical equivalence’ in one’s particular application. Recall that ϕ is the size of the interval containing all components of the underlying parameter*** w***.

Recall also that the accumulation model means all these components cluster in a ‘small’ interval around $\left(\frac{1}{T},\ldots ,\frac{1}{T}\right)$. We are therefore required to set threshold a as ‘small’ enough to match the scientifically appropriate notion of ‘small’. Similarly, ϕ is highest when one period dominates all the others. Hence, we must set b as ‘large enough’ so that one period must dominate the rest in a scientifically ‘critical’ fashion. In other words, we cannot avoid assigning precise values to the otherwise descriptive terms ‘critical’, ‘accumulation’ and ‘sensitive’, although such values can themselves be subject to sensitivity analyses.

Furthermore, we discourage generically specifying a,b to ensure a uniform distribution over the three competing ROPEs: this speciously puts the three models on an equal footing but poorly operationalizes the life-course models. In situations of very high scientific uncertainty, one palatable *generic* specification is (a,b) =(0,1), i.e. we accept the sensitive model without question. This is appropriate when we are truly ignorant about the parameters: without auxiliary ROPEs, a uniform distribution on weights implies that we believe the sensitive model. This is because $\Delta^{T}$ overwhelmingly comprises vectors whose components can be fully ranked and collectively these ‘sensitive’ vectors have prior probability 1. The posterior sensitive ROPE in this setting is itself meaningless: it vacuously has posterior probability 1 because it was given prior probability 1. Any interesting inference then hangs on whether the FCR is or is not itself the vacuous rank 1,2,.,T. In this situation, we therefore simply proceed to independently identify the FCR. Our simulations take an intermediary position. Our particular choice of (a,b)=(0.15,0.85) for this paper somewhat boosts the critical and accumulation models while preserving the natural advantage of the sensitive model. As a consequence, we placed a higher bar on posterior support for the accumulation and critical models. Conversely, because of our prior bias for the composite sensitive model, it should be accompanied by its (unbiased) FCR-model decomposition.

Despite these limitations, our proposed framework allows researchers to test central models of life-course epidemiology. We presented straightforward extensions to address some often-encountered complications in life-course modelling: e.g. causal modelling, missing data and situations involving autocorrelated errors (or nested data). The framework requires decisions to quantify the ROPEs (a, b) and also criteria by which to draw substantive conclusions from the probabilities of the rankings (Figure 2)—specifications that will put tests of life-course models on firmer scientific ground.

## Supplementary data

Supplementary data are available at *IJE* online

## Ethics approval

Add Health Study protocols were approved by the institutional review board at the University of North Carolina, approval #13–1946.

## Funding

This research was supported by R01-HD087061 and the Jacobs Center for Productive Youth Development at the University of Zurich. We use data from Add Health, a project directed by Kathleen Mullan Harris and designed by J. Richard Udry, Peter S. Bearman and Kathleen Mullan Harris at the University of North Carolina at Chapel Hill and funded by grant P01-HD31921 from the Eunice Kennedy Shriver National Institute of Child Health and Human Development, with cooperative funding from 23 other federal agencies and foundations.

## Data availability

Add Health data are available through a restricted data-use contract (see https://addhealth.cpc.unc.edu/data/#restricted-use).

## Supplementary Material

dyab073_Supplementary_Data
