# Supplementary material for: A Bayesian approach to comparing common models of life-course epidemiology
Source: Int J Epidemiol. 2021 May 10;50(5):1660–70. doi: 10.1093/ije/dyab073 (PMC8580273; doi:10.1093/ije/dyab073)
Supplement: dyab073_Supplementary_Data [file dyab073_supplementary_data.docx]

A Bayesian approach to common models of life course epidemiology

# Supplementary material

### Implicit assumptions in prior work

We elaborate on two assumptions required by Madathil et al.’s (1) approach. The first concerns the use of credible sets. To compare the three broad life course hypotheses, Madathil et al. determine whether the posterior 95% credible regions exclude or include (cover) the accumulation or critical hypotheses which are treated as points in parameter space. These points are, respectively, a) *accumulation* $w_{t}=1/T$ for all $t$, for example $\mathbf{w}=(\frac{1}{3},\frac{1}{3},\frac{1}{3})$ in the $T=3$ period situation, and b) *critical period* $w_{t}=1$ for some unique $t$, for example $\mathbf{w}=(0,0,1)$. This approach is properly implemented using multivariate credible sets, but the limitations of continuous, multidimensional credible sets create challenges in bounded parameter spaces, such as $\Delta^{T}$. Multivariate credible sets may falsely exclude the critical model, which is found on the boundary of parameter space. They may also falsely include the accumulation model if practitioners erroneously conclude that marginal coverage implies joint coverage. In fact, a multivariate set $S$ may exclude a point $p$, even while all of $P$’s lower dimensional (marginal) projections include all of $p$’s projections.

Supplementary Figure 1 provides some indication of the potential problems arising from the use of multivariate credible sets. We might try to evaluate the “red star” hypothesis – which sits in the middle of the joint parameter space - by asking whether it is credible, that is, whether it is within the multivariate credible set (two-dimensional ellipse). In Figure 1, the red star point hypothesis is not credible by this measure. If we consider the two one-dimensional credible sets represented as intervals on the axes, they are derived as having projected or “marginalized” the two- dimensional joint posterior onto each component parameter. Crucially, both these blue stars, sitting on the *x-* and *y-*axis respectively, now appear credible: they are inside both marginal credible intervals. For this and other reasons, we would ideally avoid multivariate credible sets.

Supplementary Figure 1. Two-dimensional credible set for two arbitrary parameters $(b_{1},b_{2})$ and its corresponding two univariate credible sets. The red star is a point in parameter space which is of theoretical interest. Similarly, the two black stars represent the joint hypothesis (red star) projected onto the margins. Such situations arise both in familiar Euclidian vector spaces, as well as in the probability simplex $\Delta^{T}$.

Second, the use of the density of distances from hypothetical points in parameter space requires a choice of a point hypothesis (which, ideally, should be avoided) and a choice of metric on parameter space (e.g., the Euclidean metric as opposed to the Hilbert projective metric or the Aitchison metric on the simplex). Our sensitive model decomposition is based on order relations and does not require any metric.

### Defining alternative priors

Our first methodological proposal offers a new way of choosing between accumulation, critical, and sensitive models. In contrast to our proposed strategy and that of Madathil et al. (1), a more conventional approach to model selection using Bayes factors would require explicitly specifying point mass priors on the critical and accumulation points, as well as a continuous prior over the sensitive hypothesis. As explained below, this conventional approach mandates a dramatic increase in the prior plausibility of the accumulation and critical cases, which are otherwise implausible on mathematical grounds alone. It is partly to avoid artificially distinguishing the two point hypotheses in this way and necessarily boosting their prior plausibility that we follow Madathil et al. (1). Like those authors, we choose a uniform prior over all $\Delta^{T}$, but we proceed by defining “near accumulation” or “near critical” models (not points) via two thresholds on the domain of our derived parameter $\phi$, the *range* of weights. In either case, clearly prior uniformity over $\mathbf{w}$, implemented by, for example, setting the Dirichlet hyperparameter to $\alpha=1$, actually favours the sensitive model: it does *not* imply prior uniformity over the critical, accumulation, and sensitive hypotheses. This simply reflects the mathematical fact that the accumulation and critical points occupy less of $\Delta^{T}$ and are, therefore, assigned proportionally less prior probability. In fact, interpreting the critical or accumulation hypotheses as points would imply they have zero prior and posterior probability under any continuous distribution: technically speaking, only volumes, and not individual points, have non-zero probability in this setting. Conversely, the sensitive hypothesis naturally comprises the entire volume of parameter space $\Delta^{T}$ and, hence, has 100% prior and posterior probability. Of course, a uniform prior on the weights $\mathbf{w}$ might be inappropriate in applications with substantial prior scientific knowledge. Our approach can accommodate such applications where previous data or theory indeed support a critical or accumulation model *a priori*: by calibrating the Dirichlet hyperparameter, the scientist could introduce prior bias of the desired strength towards either the accumulation model ($\alpha>1$) or the critical model ($\alpha<1$).

Our second method decomposes the sensitive model, yielding an inference about which measurement occasions are more important for the outcome. It should be noted that, unlike the case of model adjudication discussed in the previous paragraph, each constituent full ranking of the sensitive hypothesis, say $3|1|2$ or $3|2|1$, *occupies equal volume of* $\Delta^{T}$. In contrast to model adjudication via $\phi$, the uniformity of $p(\mathbf{w})$ is, therefore, indeed preserved. In particular, each full ranking is uniformly assigned $1/T!$ prior probability. A general partial ranking is assigned prior probability $k/T!$, where $k$ is the number of underlying full rankings that comprise the partial ranking. For example, partial ranking $2|1,3$ has $k=2$ and prior equalling $2/3!$ because $2|1,3:=2|1|3 or 2|3|1$. In applications where there are clear competing scientific theories about the precise sensitivity profile *a priori*, it may be possible to use confirmatory methods suggested in the literature on so-called informative hypotheses (2–5).

### Alternative ROPEs

Supplementary Figure 2: The preimages of [0, *a*], (*a, b*) and [𝑏, 1] as polygons in the case of three time points. As the figure shows, our ROPES are not entirely free from criticism. For example, consider the thresholds of *a =* 0.2 and *b =* 0.8, the interval of 𝜙 that should be considered for the critical period hypothesis is [0.8, 1]. The ROPE here is a polygon with vertices at 𝑤 = {(1.0, 0.0, 0.0), (0.8, 0.0, 0.2), (0.8,0.2,0.0), (0.8667,0.0667,0.0667)}. Interestingly, the point 𝑤 = (0.85, 0.075, 0.075) is not in the ROPE (𝜙 = 0.77); while the point 𝑤 = (0.8, 0.2, 0.0) is in the ROPE. However, the former sample apparently supports a critical period hypothesis. As with any ROPE, readers must decide for themselves whether the geometry of these particular ROPES is acceptable. In simulations our ROPEs, which have a pleasing connection to the range or variability $\phi$ of weights, had satisfactory statistical performance.

While we have used 𝜙 to create ROPEs for omnibus models, there are many other reasonable and intuitive ways to create ROPEs. Such alternative constructions should be conceptually analogous to ours, differing only in their precise geometry. They may nonetheless give some intuition for our choice of 𝜙. For example, rather than $\phi<a$, the accumulation ROPE could be taken as the set of $\mathbf{w}$ whose components are all within some small distance $a'$ of $(\frac{1}{T},..,\frac{1}{T})$. Similarly, rather than using $\phi>b$, the omnibus critical ROPE could be taken as the set of$\mathbf{w}$ for which any component^[[1]](#footnote-1)^ exceeds threshold $b'$. The sensitive omnibus ROPE is then just the complement of these two ROPEs, i.e. the rest of $\Delta^{T}$. Like those based on $\phi$, these alternative ROPEs can also be used when we are uncertain *a priori* about which specific critical model may be true. (They also offer a multiple comparisons adjustment.) These alternative ROPEs can fairly be compared with ours when $\left( a^{'},b^{'} \right)$ is chosen to satisfy

$$p\left( \phi<a \right)=p(\mathbf{w}\in\Delta^{T}:\forall i |w_{i}-1/T|<a')$$

$p\left( \phi>b \right)=P\left( \mathbf{w}\in\Delta^{T}:\exists i w_{i}>b' \right)$.

Note that the latter has strictly greater probability than any one critical model *i*. This is because the monotonicity of probability gives $P\left( \mathbf{w}\in\Delta^{T}:\exists i w_{i}>b^{'} \right)\geq p\left( \mathbf{w}\in\Delta^{T}:w_{i}>b^{'} \right)$ where *i* is free in the former and fixed in the latter. It is obviously also smaller than $T \times p\left( \mathbf{w}\in\Delta^{T}:w_{i}>b^{'} \right)$. We numerically solved the two equations above in order to examine the behavior of these alternative ROPEs in our simulations. The ensuing posterior inferences were qualitatively alike, demonstrating some robustness to the exact geometry of the ROPEs.

### mRNA preprocessing

FASTQ sequencing data was transformed into counts using the STAR aligner. ENSG gene identifiers were then mapped to a Gene Symbol and counts for the same gene symbol were summed (i.e., summing over alternative transcripts/versions of the same gene). Note that in many cases multiple distinct ENSG identifiers may be ascribed to a single gene symbol, and in other cases, there may be no gene symbol to map to. In the latter case, the ENSG identifier is retained.

Starting from 59068 gene ids, we removed haemoglobin genes and genes with no HUGO ID, leaving 55772. We then removed genes with insufficiently large counts to be retained in a statistical analysis, resulting in 8484. The latter was performed in edgeR, using filterByExp, using a filtering strategy described by Chen & Smyth (2016). Roughly speaking, this strategy keeps genes that have at least 10 reads in a worthwhile number of samples.

We reference-gene normalized the count data, yielding log Transcripts Per Million (TPM). This involves dividing by the mean count over the 11 housekeeping genes identified by Eisenberg and Levanon (2013) (2013, Table 1): C1orf43, CHMP2A, EMC7, GPI, PSMB2, PSMB4, RAB7A, REEP5, SNRPD3, VCP, VPS29. See (6) for more details.

# REFERENCES

1. Madathil S, Joseph L, Hardy R, Rousseau M-C, Nicolau B. A Bayesian approach to investigate life course hypotheses involving continuous exposures. International Journal of Epidemiology. 2018;47(5):1623–35.

2. Gu X, Mulder J, Deković M, Hoijtink H. Bayesian evaluation of inequality constrained hypotheses. Psychological Methods. 2014;19(4):511.

3. Klugkist I, Kato B, Hoijtink H. Bayesian model selection using encompassing priors. Statistica Neerlandica. 2005;59(1):57–69.

4. Mulder J, Hoijtink H, Klugkist I. Equality and inequality constrained multivariate linear models: Objective model selection using constrained posterior priors. Journal of Statistical Planning and Inference. 2010;140(4):887–906.

5. Mulder J, Olsson-Collentine A. Simple Bayesian testing of scientific expectations in linear regression models. Behavior Research Methods. 2019;1–14.

6. Cole, SW, Shanahan, MJ, Gaydosh, L., & Harris, KM. In press. Inflammatory and antiviral gene expression in Add Health: Molecular pathways to social disparities in disease emerge by young adulthood. *PNAS*

1. Strictly speaking a *unique* component $\exists!i w_{i}>b'$. Uniqueness preserves the interpretation as a critical hypothesis, and is ensured whenever $b^{'}>0.5$, i.e. high enough that multiple components of the same point $\mathbf{w}$ do not simultaneously exceed $b'$. [↑](#footnote-ref-1)
